# The Relationship between Employee Risk Communication and Non-Adaptive Evacuation Behavior in Chinese Hazardous Chemical Companies: The Mediating Role of Emotional Exhaustion and Risk Perception

**DOI:** 10.3390/ijerph191811432

**Published:** 2022-09-11

**Authors:** Zilin Yang, Xinping Wang, Chang Su, Boying Li

**Affiliations:** 1 School of Management, Xi’an University of Science and Technology, Xi’an 710054, China; 2 School of Safety Science and Engineering, Xi’an University of Science and Technology, Xi’an 710054, China; 3 School of Political Science and International Relations, Tongji University, Shanghai 200092, China

**Keywords:** non-adaptive evacuation, risk communication, emotional exhaustion, risk perception, SEM

## Abstract

Non-adaptive evacuation behavior refers to a safety risk that cannot be disregarded throughout the evacuation process. In order to lower the risky behavior of evacuation, enhancing people’s psychological states and behaviors plays a significant role. This study developed a conceptual model connecting risk communication and non-adaptive evacuation behavior by analyzing the interaction between risk communication, risk perception, emotional exhaustion, and non-adaptive evacuation behavior. The structural equation model was adopted to analyze the 557 questionnaires collected, by which the findings demonstrated that risk communication has a negative impact on non-adaptive evacuation behavior, which is also indirectly affected by risk perception and emotional exhaustion. With the aim to prevent non-adaptive evacuation behavior during the evacuation process, enterprises and organizations can adjust the behavior level, psychological state, and physiological condition of individuals from the perspectives of risk communication, risk perception, and emotional exhaustion.

## 1. Introduction

China has been the global leading country in the chemical industry since 2010. The chemical sector in China contributes to 40% of the global output. Chinese chemical companies have been extensively expanding in the recent past. Chemical businesses are significantly affected by fire accidents or other emergencies due to their complex production processes, as well as flammable, explosive, toxic, and corrosive materials, compared with other types of businesses. A previous study reported 5207 hazardous chemical leakage accidents (HCLAs) and 157 evacuations associated with HCLAs in China between 2009 and 2018 [1]. Approximately 20% of hazardous chemical accidents require evacuation of nearby residents due to the fast-burning speed, wide range coverage, harmfulness to the human body, difficulty in control, and high-risk factors. In addition, there is no effective standard regulations for the safety distance between chemical plants and residential areas in China [2]. Currently, the number of chemical plants in China is two-fold that of other developed countries [3]. This implies that the loss associated with accidents in chemical plants is severe and the social impact is significant. Unsafe production and insufficient emergency response capacity of hazardous chemical plants are the primary causes of natural, technical, or artificial accidents [4]. Risks can only be effectively avoided from the perspective of enterprises by strengthening supervision and safe production due to the indefinite nature of accidents. However, improving emergency response mechanisms and improving their safe behavioral capabilities can reduce unnecessary losses for accidents caused by natural factors such as earthquakes and typhoons. Studies report that accident outcomes can be reduced by establishing risk management systems in advance, and guiding individuals to take effective protective actions such as evacuation and sheltering [5]. A major challenge for China’s emergency management industry is to rapidly evacuate the public to safe areas during emergencies such as fires and earthquakes that affect hazardous chemical facilities while minimizing casualties and losses related to the disasters. The process of evacuating a crowd in an emergency is a complex, and it is important to understand human behavioral activities from the perspective of complexity science to effectively evacuate the crowd and avoid cascading effects [6].

People’s behavior is apparent, whereas their mental activity is implicit, and the internal mental activity invariably controls the outward behavior. People are intensely focused on the emergency when it occurs. This increased attention alters the emergency response and evacuation process. Individuals’ cognitive, emotional, and physiological resources are depleted as a result of the ambiguity, urgency, and unconventionality of emergencies. Moreover, external circumstances significantly affect the mental and physiological resources, resulting in negative emotions such as panic and stress. Non-adaptive evacuation behaviors, such as herding behavior [7], avoidance behavior [8], and competitive behavior [9]. Individuals may exhibit behaviors that hinder or affect evacuation efficiency due to the modified psychological state and disaster stress, which may ultimately endanger others and minimize group evacuation [10,11].

Collective decision-making from hazard warning information is significantly modulated by psychological and social environmental factors [12]. Koo reported that the existence of unanticipated risks can abruptly result in mental disorientation caused by mounting emotions and a longer evacuation time [13]. In addition, physical exhaustion during an evacuation can prolong the evacuation duration [13]. Findings by Wang et al. indicate that panic affects people’s decision-making processes and their emotions [14]. High levels of panic increases susceptibility of people to danger, reduces effectiveness of evacuation, and increases risk of abnormal conduct in the crowd. People require timely risk information during emergencies to help them navigate their way out of the crisis. Spreading risk information through social networks ensures that more people receive the warning messages [15]. Risk perception, which is defined as the subjective evaluation of an emergency’s unfavorable effects by a person mainly through the examination of communication data, is an essential factor in determining behavior during evacuation [16]. Understanding the psychological and behavioral state of individuals can significantly improve the effectiveness of emergency evacuation and lower personal as well as property losses. Individuals significantly affect evacuation behavior, and various ways can be used to reduce the stress caused by emergency events to people. The aim of this study was to explore the effects of non-adaptive evacuation behavior based on the risk communication ability, risk perception, and emotional exhaustion levels of the parties involved in an emergency situation.

The relationships of risk communication, risk perception, emotional exhaustion, and non-adaptive evacuation behavior were explored through literature review, and research hypotheses and conceptual model were formulated. Data were through an actual questionnaire, and the established conceptual model was verified using SPSS and Amos software. The first part of this paper is the Introduction, which describes the background of the research and the research objective. The second section comprises formulation of the research hypotheses, the possible relationship between the variables were determined through literature review, and a conceptual model of the research was constructed. The third section comprises the research methods, which mainly presents the questionnaire survey used to obtain data in this research, including designing of the questionnaire, the study site, the distribution strategy, and the number of questionnaires distributed.

## 2. Theoretical Basis and Research Hypothesis

### 2.1. Risk Communication and Non-Adaptive Evacuation Behavior Research Hypothesis

Individuals’ cognitive capacities are not impaired when they face an unpredicted catastrophe. On the contrary, these individuals can effectively handle limited information [17]. Individuals should rely on both common-sense evacuation and highly credible information obtained from a variety of external sources to quickly assess the severity of the event and escape from the danger. The reliable information includes guidance on emergency evacuation, orders from the leader of the field staff, and communication with peers. The occurrence of disasters affects people’s emotional state, which in turn affects their behavior and attitudes. Residents rely on highly viable information from various sources in addition to their catastrophe knowledge in order to make the right judgements on the likelihood and severity of a disaster [18]. The process of conveying salient risk information is known as risk communication [19], and people mainly rely on individuals with more understanding to obtain this type of information. The National Research Council defined risk communication as “the interactive process of sharing information and ideas among individuals, groups, and institutions” [20]. In this study, we focused on the risk information transmission between risk information providers (hazardous chemical enterprises and emergency command experts) and evacuees due to the limitation of two-way information dissemination in emergencies. Risk communication can alter an individual’s awareness through the interchange and transmission of risk information [21], ultimately affecting the person’s conduct. People take their time to research more pertinent information before making decisions owing to the uncertainty associated with emergencies. The public makes sound decisions about risks because related information is readily available, and risk communication enhances decision-making and risk-sharing. Therefore, a high-quality risk communication process between relevant parties and the evacuees ensures improved assistance during emergencies and helps people to overcome adverse emotions such as anxiety and fear associated with sudden public emergencies. Moreover, it provides a basis for formulating the evacuation and escape process, and reduces accidents that can be caused by non-adaptive psychology during evacuation. The following hypotheses were proposed in the study according to these findings.

**Hypothesis** **1** **(H1).** *Risk communication has a negative effect on non-adaptive evacuation behavior.*

### 2.2. Emotional Exhaustion’s Mediating Function

People commit more time, energy, and mental and physical resources to deal with the outcomes of an emergency due to the unpredictability nature and accidents that occur during emergencies. The theory of uncertainty management [22] indicates that uncertainty weakens a person’s sense of self, diminishes their sense of control over and predictability of their environment, and reduces the sense of security. Furthermore, uncertainty promotes disgust, makes individuals uncomfortable, and promotes strong negative emotions. The notion of resource conservation [23] reports that people have a propensity to acquire, defend, and conserve resources, and any loss of resources makes them anxious and stressed out. The uncertainty of unexpected events and other factors cause a state of high physiological and psychological tension in individuals resulting in depletion of their cognitive, emotional, and physiological resources, or emotional exhaustion, ultimately affecting people’s attitudes, behaviors, and performance [24]. People cannot deal with the unexpected circumstance with their typical problem-solving strategies during emergencies because of the extreme stress that occurs when the social disruption caused by the disaster surpasses a person’s coping capacity. As a result, people present with emotional responses and are highly likely to develop non-adaptive psychological traits such as panic and herding behavior. Under emergencies, people disregard the group interest and engage in non-adaptive behaviors such as selfishness, small group behavior, and panic behavior to find survival opportunities [25]. These behaviors can reduce evacuation efficiency, lengthen time taken in evacuation and result in expanded consequences as the wave of panic spreads through the crowd, the degree of emotional exhaustion increases, and the level of concentration decreases [26]. Learning to control irrational emotions and maintain composure during a crisis is essential to increase efficiency of individual emergency response because there is a limit to the level of rationality that an audience can maintain in an emergency. The level of fear and panic associated with emergencies is more harmful to individuals than the risk itself. Effective communication alleviates the psychological and cognitive consequences of disasters on people [27]. Immediate risk communication is essential to reduce the emotional exhaustion caused by the uncertainty of a crisis [28]. Timely risk communication ensures that individuals receive the relevant risk information, alleviates the information shortage related with emergencies, enhances effective understanding of the accident, and enables individuals to escape from the crisis through guidance information, thus alleviating their psychological and physical stress.

These findings indicate that risk communication can effectively provide risk information and alleviate the unsafe behaviors caused by emotional exhaustion in individuals. In addition, the depletion of physiological and psychological resources during emergencies leads to a series of unsafe behaviors that increase the risk of individuals during evacuation. The following hypotheses were proposed based on these findings.

**Hypothesis** **2** **(H2).** *Emotional exhaustion has a mediating role in risk communication and nonadaptive evacuation behaviors.*

**Hypothesis** **2a** **(H2a).** *Risk communication has a negative effect on emotional exhaustion.*

**Hypothesis** **2b** **(H2b).** *Emotional exhaustion has a positive effect on non-adaptive evacuation behavior.*

### 2.3. Risk Perception’s Mediating Function

Risk perception is impacted by a person’s characteristics, the availability of warning information, and time. Risk perception is defined as the cognitive capacity to identify risk in various circumstances [29]. People are guided away from their current environment by elements including stimuli, risk information, and other factors that influence the creation of risk perceptions. Notably, development of risk perceptions enhances protective behavior [30]. The protective motivation theory states that people choose protective actions after evaluating the associated risk. Previous studies demonstrate that risk perception significantly modulates individual protective behavior [31]. Risk warning information provided during emergencies can improve the public’s perception of the accident and promote emergency protective actions [32]. Effective information dissemination is essential for information management and risk communication strategies. Positive information alleviates negative emotions such as anxiety and fear associated with accidents. Positive public risk information and negative private risk information are positively correlated with residents’ evacuation [33]. Moreover, negative fear information stimulates protective actions and increases risk awareness [34]. Effective conveying of accurate disaster information to residents through reliable channels increases the level of disaster risk awareness, increases safety knowledge and skills, improves safety behaviors, and enhances individuals’ risk perceptions, which ultimately influence their behavioral decisions [35]. Therefore, higher safety awareness and alertness of individuals during emergencies is associated with stronger risk perception ability, and enhances behavioral characteristics for safe evacuation [36]. Individuals pay more attention to the safety signs and guidance information around them, adopt preventive behaviors, reasonably use risk information guidance for a safe evacuation, avoid non-adaptive behaviors related with information shortage, and improve evacuation efficiency to ensure they are safe.

These previous studies indicate that depletion of physiological and psychological resources caused by emergencies increases the risk of unsafe behaviors among individuals during emergency evacuation. Notably, risk communication effectively provides key information on accidents and alleviates the emotional exhaustion among individuals. The following hypotheses were proposed based on these findings:

**Hypothesis** **3** **(H3).** *Risk perception has a mediating role in risk communication and non-adaptive evacuation behaviors.*

**Hypothesis** **3a** **(H3a).** *Risk communication has a positive effect on risk perception.*

**Hypothesis** **3b** **(H3b).** *Risk perception has a negative effect on non-adaptive evacuation behavior.*

### 2.4. Chain Intermediates’ Function

Sufficient flow of risk information is essential for effective communication between subjects. Information asymmetry exists because of the subjectivity of public cognition and promotes negative emotions about the effect of risk communication [37]. Previous findings show that emotions have a direct impact on individual judgment and decision-making [38]. Perceived risk at work can be improved through high-quality information communication between employees about the risk and a swift decision-making process that prioritizes safety [39]. The JD-R’s Health Impairment Process indicates that specific resources (such as time and energy) are required for conveying risk information, but these resources cause emotional exhaustion over time [40]. Resource depletion significantly affects safe behavioral performance of an individual. An individual’s cognitive level, social, and personal abilities complement each other and contribute to safe and efficient performance [41]. A chain relational model of “risk communication-risk perception-emotional exhaustion-non-adaptive evacuation behavior” was developed using the functions described above. The model indicates that risk communication enhances an individual’s level of risk perception, increases their level of alertness, and improves risk identification ability, as well as helps them overcome the emotional exhaustion caused by emergencies. These changes decrease the frequency of non-adaptive evacuation behaviors. The following hypothesis was proposed based on these findings.

**Hypothesis** **4** **(H4).** *Risk perception and emotional exhaustion play a chain-mediated role in risk communication and maladaptive evacuation behavior.*

**Figure 1:**
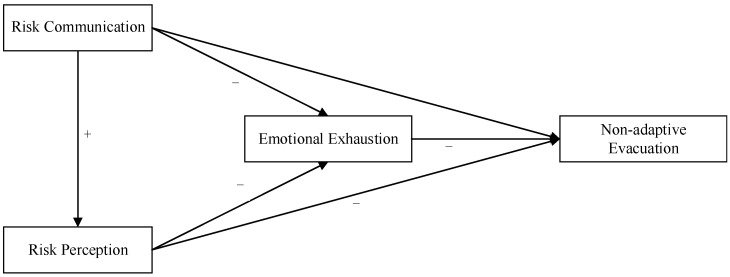
Conceptual model. “+” means there is a positive relationship. “−” means that there is a negative relationship.

Therefore, based on the research hypothesis proposed above, the conceptual model of this study is formed, as shown in Figure 1. 

## 3. Research Methodology

### 3.1. Variable Measurement

Based on already available and validated scales, this questionnaire examines the relationship between emotional exhaustion, risk perception, risk communication, and nonadaptive evacuation behaviors. The items were measured based on the contingency’s context, with the measures of risk communication primarily referring to Edwards’ ten-item scale [42], the measures of employee emotional exhaustion primarily referring to Watkins’ scale [43], the measures of risk perception primarily referring to Slovic’s risk perception scale [29], and the measures of non-adaptive evacuation behavior primarily referring to Wang’s scale [11]. Gender, age, education, working years, occupation, and emergency evacuation experience were all included in the demographic data. A total of 35 items made up the measuring questions, which used a five-level Likert scale to measure the variables.

### 3.2. Basic Details

Before the questionnaire was formally distributed, this study conducted a trial distribution in April 2021 to assess the validity and reliability of the aforementioned factors and to modify the questionnaire to account for unclearly worded items. The official distribution of the questionnaire will be carried out in September 2021. Questionnaires will be distributed to hazardous chemical enterprises in Shanghai, Shaanxi, and Shanxi, mainly including construction enterprises, hazardous chemical enterprises, etc. The respondents are all employees of hazardous chemical enterprises. Certain safety production education and training, and all volunteered to participate in this investigation. This study was approved by the ethics board. The survey is mainly based on a combination of on-site distribution and electronic questionnaires. A total of 677 questionnaires were collected from the end of April to the end of September 2021. After excluding invalid questionnaires such as incomplete items and too short filling time, a total of 557 valid samples were collected. The basic information about the samples is shown in Table 1.

## 4. Results of Data Analysis

### 4.1. Reliability and Validity Test

The reliability analysis was mainly conducted by SPSS 26.0 (IBM, Armonk, NY, USA), and Cronbach’s alpha coefficient was used to test the reliability of each variable in this paper. The Cronbach’s alpha values of risk communication, emotional exhaustion, risk perception, non-adaptive evacuation behavior, and the questionnaire overall were all greater than 0.7 (as shown in Table 2), indicating that the questionnaire has good reliability. The two main areas of investigation in terms of validity testing were content validity and structural validity. The questionnaire’s KMO value is 0.902, which is greater than 0.8, and Bartlett’s spherical test value is 0.000, which is suitable for exploratory factor analysis. All scales are from significant international journal literature, and they all have good content validity. The structural validity of the questionnaire needs to be conducted by exploratory factor analysis and validation factor analysis. And all convergent reliability (AVE) is greater than 0.5 indicating high convergent validity, and all component reliabilities (CR) were greater than 0.8, indicating sufficient internal consistency of the constructs. As a result, it shows that the questionnaire has a high degree of validity and reliability.

### 4.2. Analysis of Correlations between Variables

The correlation between the variables was confirmed using the Spearman correlation coefficient. The correlation of risk communication, emotional exhaustion, risk perception, and non-adaptive evacuation behavior are the primary variables in this paper, as shown in Table 3 below. There is negative correlation (*p* < 0.01), substantial positive association between risk communication and risk perception, a significant positive relationship between emotional exhaustion and non-adaptive evacuation behavior, and a significant negative relationship between non-adaptive evacuation behavior. As a consequence, the assumptions H1, H2a, H2b, H3a, and H3b can be preliminarily verified.

### 4.3. Model Fit Test

The fit indicators and accompanying criteria are provided in Table 4 for the structural equation model of the link between risk communication and non-adaptive evacuation behavior. The three most important evaluation indicators—the RMSEA, CFI, and χ^2^/df—exceed the threshold. Overall, it can be observed that the model delivers the required outcomes and is totally acceptable, the model test succeeds, and all sorts of fit indicators fulfill the evaluation criteria, demonstrating the model’s good fit.

### 4.4. Results of the Hypothesis Test

**Figure 2:**
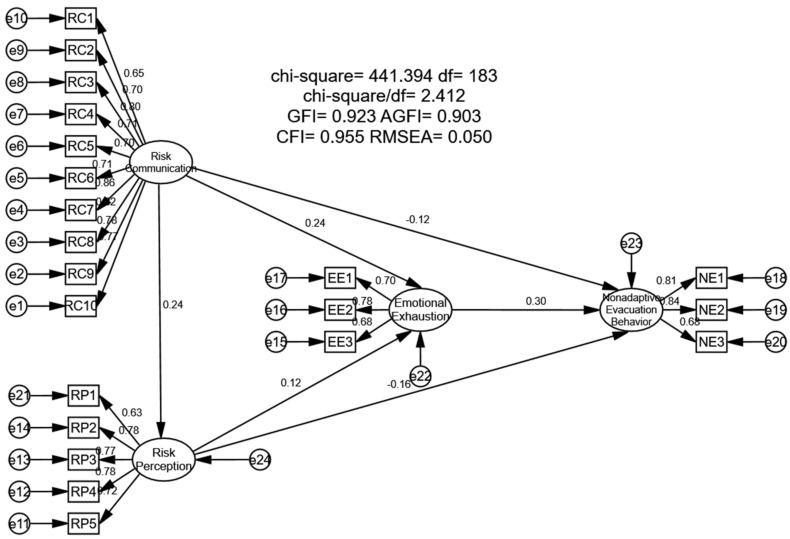
Structural equation model analysis results.

Figure 2 shows the path parameters of the influence mechanism between risk communication and non-adaptive evacuation behavior using AMOS 26.0 ( IBM, Armonk, NY, USA) evaluate the model’s hypotheses. According to Figure 2 and Table 5, the path coefficient between risk communication and non-adaptive evacuation behavior is −0.12 (*p* < 0.05), indicating a negative relationship between risk communication and non-adaptive evacuation behavior; the path coefficient between risk communication and risk perception is 0.24 (*p* < 0.001), indicating a more significant positive relationship between risk communication and risk perception; the path coefficient between risk communication and non-adaptive evacuation behavior is 0.24 (*p* < 0.001), indicating a negative relationship between risk communication and emotional exhaustion. The path coefficient between emotional exhaustion and non-adaptive evacuation behavior was 0.30 (*p* < 0.001), indicating a positive relationship between role width self-efficacy and role overload. The path coefficient between risk perception and non-adaptive evacuation behavior was −0.16 (*p* < 0.01), indicating a significant negative relationship between risk perception and non-adaptive evacuation behavior. As a consequence, the paper’s Hypotheses H1, H2a, H2b, H3a, and H3b are valid.

### 4.5. Mediation Test

In order to confirm the mediating function and the indirect impacts of risk perception and emotional exhaustion on non-adaptive evacuation behavior, a 5000-time Bootstrap test was performed in this study. The results are shown in Table 6. At the 95% confidence level, the Bias-Corrected and Percentile confidence intervals of each mediation pathway do not include 0, indicating that risk perception and emotional exhaustion have a significant effect on the relationship between risk communication and non-adaptive evacuation behavior. The mediation model is established. Therefore, the mediating effect of role overload and role-width self-efficacy in each path is a partial mediating effect, so Hypotheses H2, H3, and H4 are established.

## 5. Discussion of Empirical Results

According to the aforementioned result, the conceptual model proposed in this work has already undergone preliminary validation. The conceptual model’s hypotheses are all validated, and the path coefficients are all statistically significant, demonstrating that, to varied degrees, risk communication, risk perception, and emotional exhaustion all affect non-adaptive evacuation behavior directly and indirectly.

(0) With path factor loadings of −0.155 and −0.116, respectively, risk perception and risk communication have comparable degrees of influence on non-adaptive evacuation behavior. With a path factor loading of 0.301, emotional exhaustion has the greatest impact on non-adaptive evacuation behavior. The study’s Hypotheses H1, H2b, and H3b are all supported by the data, demonstrating that non-adaptive evacuation behavior is influenced by risk communication, risk perception, and emotional exhaustion. The results show that emotional exhaustion is a significant factor influencing people’s risky behavior during the evacuation and has the greatest impact on non-adaptive evacuation behavior. Therefore, in order to decrease the negative effects of emotional exhaustion, each organization should pay more attention to the physical and mental health of its employees, assess how emotional exhaustion affects employee safety behavior, and restrict non-adaptive evacuation behavior to lessen the negative role of emotional exhaustion.
(1) The mediating role of risk perception and emotional exhaustion is highlighted in Hypotheses H2, H3, and H4.

The validity of Hypothesis H2 implies that effective risk communication is essential in influencing people’s non-adaptive evacuation behaviors because emotional exhaustion plays a mediating role in the relationship “risk communication—emotional exhaustion—non-adaptive evacuation behaviors”. This demonstrates how effective risk communication in times of crisis can modify people’s emotional exhaustion levels, effectively lowering non-adaptive evacuation behavior. Risk communication can improve non-adaptive evacuation behavior by influencing risk perception, according to the hypothesis “risk communication—risk perception—non-adaptive evacuation behavior”. According to Hypothesis H3, people’s non-adaptive evacuation behavior is significantly influenced by their perception of risk. This suggests that good risk communication may reduce non-adaptive evacuation behaviors by increasing people’s perceptions of risk, hence assisting in the achievement of the goal of safe evacuation. According to H4, risk perception and emotional exhaustion all play mediating roles in this process. People with stronger risk perception skills are better able to recognize and apply risk information in high-risk communication, lessen worry and uncertainty brought on by a lack of knowledge, and address emotional exhaustion in others in an efficient manner. In turn, this reduces the maladaptive action of evacuation brought on by maladaptive psychology. This implies that effective risk communication has the power to alter people’s cognitive and psychological states, which in turn have an impact on their behavior.

(2) It is concluded that, in the event of an emergency event, increasing the level of risk communication can give people specific risk information, support them in identifying risks, and provide them with guidance on how to proceed based on the findings of the aforementioned study and a thorough analysis of the relationship between risk communication, risk perception, and emotional exhaustion. Individuals’ non-adaptive evacuation behavior can be improved by emotional exhaustion and risk perception. Therefore, organizations and organizations must pay attention to each individual’s psychological health level as well as their safety behavior. They also need to develop their capacity for risk communication and strengthen their ability to provide risk information assistance.

## 6. Suggestions

(1) Management Implications:

For risk communication, the key to enhancing the risk communication ability of all parties is learning how to provide accurate and effective guidance information and improving the coordinating skills of emergency commanders. To determine the accident’s occurrence, the affected area, and the extent of the event, as well as to transmit risk information to the trapped people and lay the groundwork for the subsequent direction of people to escape the predicament, it is necessary for the emergency response command center crew to conduct thorough research and judgment on risk information. For individuals, to properly analyze and handle risk information, people need to have strong communication and discrimination abilities. During emergencies, people frequently feel anxious, tense, and other undesirable emotions. The organization of various safety training can help reduce individual stress, feel better emotionally, and pay better attention in situations. This will help people pay closer attention to safety information, avoid overinterpreting outside information, and actively participate in pertinent education, training, and evacuation drills to enhance their safety behavior and psychological preparedness. As a result, there will be fewer delays in evacuation because of panic in the event of a disaster.

For risk perception, risk education can increase public capacity, foster risk awareness and vigilance, and improve people’s ability to evaluate risks’ likelihood of occurring and projected severity, empowering them to act effectively and promptly to avoid potentially fatal circumstances. Secondly, to simulate accident outcomes and experience the seriousness of accident risks, accident education can be carried out by showing pertinent accident movies. This will increase their level of risk perception. For the public to readily find warning information and to improve risk warning capabilities, it is essential to strengthen the development of safety warning information facilities and to ensure that safety warning signs are hung in the proper areas.

For emotional exhaustion, the depletion of one’s own physiological and psychological resources has a significant effect on behavior in emergency situations. The psychological wellbeing of employees should constantly be taken into consideration by units, who should also set up psychological consultation rooms, schedule frequent psychological therapy, and go through training.

(2) This study has several restrictions. Firstly, this study distributes questionnaires to hazardous chemical companies. Because of the particularity of the industry, employees in hazardous chemical companies have greater training in emergency evacuation, safe escape, etc. There are certain restrictions on data collecting; therefore, future research may need to broaden it to validate if the results apply to other contexts. The results of this study can be made more convincing in future research by simulating emergency evacuation scenarios through situational simulation experiments, using scales combined with eye movement and EEG experiments to measure them together. Additionally, using scales in this study to measure changes in an individual’s emotional exhaustion during emergencies at the physical and psychological levels is subjective. Finally, although this study focused on how risk communication, risk perception, and emotional exhaustion affect people’s behavioral reactions to emergencies, data collection does not account for some organizational factors and distinctive personnel characteristics that can influence employees’ attitudes and perceptions about crises. As a result, some uncontrolled confounding factors may be present in the data analysis. Future research should concentrate on removing the influence of other variables and taking into account how relevant variables affect non-adaptive evacuation behaviors.

## 7. Conclusions

This study mainly discusses the connection between risk communication and non-adaptive evacuation behavior. Through qualitative analysis of the possible relationship between risk communication and non-adaptive evacuation behavior, the proposed conceptual model is tested quantitatively. The findings demonstrate that raising the level of risk communication can significantly reduce people’s risky behavior during evacuation. Three significant elements that influence non-adaptive evacuation behavior are discovered in addition to the mechanism of action between risk communication and this behavior.

The results show that risk communication and risk perception have a negative impact on non-adaptive evacuation behavior, but emotional exhaustion has a positive impact. Risk perception and emotional exhaustion act as a chain mediating factor. These findings offer recommendations for strategies to enhance risk communication, increase risk perception skills, diminish emotional exhaustion, and ultimately limit risky behavior. Therefore, non-adaptive evacuation behavior can be improved from three aspects: risk communication, risk perception, and emotional exhaustion.

## Figures and Tables

**Table 1:** Basic details.

Item	Category	Proportion (%)	Item	Category	Proportion (%)
sex	male	63.6	educated	Level Junior high school and below	10.1
	female	35.4		high school	19.9
length of service	<1 year	10.8		college	27.5
	2–5 years	10.7		bachelor	39.5
	6–10 years	20.5		Master degree and above	3.1
	≥10 years	49	job level	staff	47.6
emergency evacuation experience	yes	83.7		grassroots management	26.2
	no	16.3		middle management	20.8
				top management	5.4

**Table 2:** Reliability and validity test.

Variable	Quantity	Cronbach’s α	Overall Cronbach’s α	CR	AVE
Risk Communication	10	0.928	0.789	0.950	0.604
Emotional Exhaustion	3	0.759		0.863	0.678
Risk Perception	5	0.843		0.889	0.615
Non-adaptive Evacuation	3	0.818		0.892	0.734
Bartlett test of sphericity			KMO	0.902	
			Degrees of freedom	210	
			Sig	0	

**Table 3:** Correlation analysis of variables.

	Risk Communication	Emotional Exhaustion	Risk Perception	Non-Adaptive Evacuation
Risk Communication	1			
Emotional Exhaustion	−0.223 **	1		
Risk Perception	0.218 **	−0.146 **	1	
Non-adaptive Evacuation	−0.203 **	0.292 **	−0.211 **	1

** At the 0.01 level (two-tailed), the correlation is significant.

**Table 4:** Model fit test.

Test Statistic		Judgment Criteria and Critical Values	Fitting Results	Fitting Degree
Absolute Fit Index	χ^2^	The smaller the better	441.394	Well
	χ^2^/df	1–3	2.412	Well
	RMSEA	<0.08	0.050	Well
	GFI	>0.90	0.923	Well
Relative Fit Index	IFI	>0.90	0.955	Well
	TLI	>0.90	0.948	Well
	CFI	>0.90	0.955	Well
Parsimony Index	PNFI	>0.50	0.806	Well
	PGFI	>0.50	0.731	Well

**Table 5:** Direct action checklist.

Path	Factor Loadings		Parametric Significance Estimation			Conclusion
	Std.	Unstd.	S.E.	C.R.	P	
Risk Communication→Risk Perception	0.236	0.180	0.037	4.902	***	H3a √
Risk Communication→Emotional Exhaustion	−0.243	−0.197	0.042	−4.634	***	H2a √
Risk Perception→Emotional Exhaustion	−0.125	−0.132	0.056	−2.345	*	
Risk Communication→Non-adaptive Evacuation Behavior	−0.116	−0.128	0.054	−2.381	*	H1 √
Risk Perception→Non-adaptive Evacuation Behavior	−0.155	−0.224	0.072	−3.099	**	H3b √
Emotional Exhaustion→Non-adaptive Evacuation Behavior	0.301	0.408	0.076	5.382	***	H2b √

√ means hypothesis is valid. * means *p* < 0.05. ** means *p* < 0.01. *** means *p* < 0.001.

**Table 6:** Mediation test.

Influence Path	Effect Value	Multiplying Coefficients		Bootstrap			
				Bias-Corrected		Percentile	
		SE	Z	Lower	Upper	Lower	Upper
Risk Communication—Emotional Exhaustion—Nonadaptive Evacuation Behavior	−0.080	0.025	−3.200	−0.139	−0.041	−0.133	−0.037
Risk Perception-Emotional Exhaustion-Nonadaptive Evacuation Behavior	−0.040	0.018	−2.222	−0.082	−0.013	−0.080	−0.011
Risk Communication—Risk Perception—Non-Adaptive Evacuation Behavior	−0.054	0.027	−2.000	−0.120	−0.010	−0.114	−0.008
Risk Communication—Risk Perception—Emotional Exhaustion—Nonadaptive Evacuation Behavior	−0.010	0.005	−2.000	−0.024	−0.002	−0.023	−0.001

## Data Availability

The data that support the findings of this study are available from the corresponding author upon reasonable request.
